# Computed Tomography Evaluation of Morphological Types of Femoral Trochlear Dysplasia in Small-Breed Dogs—A Retrospective Study

**DOI:** 10.3390/vetsci12010049

**Published:** 2025-01-12

**Authors:** Radka S. Garnoeva

**Affiliations:** Department of Veterinary Surgery, Faculty of Veterinary Medicine, Trakia University, 6000 Stara Zagora, Bulgaria; dr.garnoeva@abv.bg

**Keywords:** small dog breeds, computed tomography, trochlear dysplasia types, prevalence, medial patellar luxation

## Abstract

Abnormal trochlear morphology is one of the most important factors for patellar luxation occurrence in dogs, yet no studies have investigated its prevalence in the general population. This retrospective computed tomography study was designed to evaluate the trochlear groove morphology in four small dog breeds and the prevalence of trochlear dysplasia types according to Déjour’s classification depending on the breed, sex, and medial patellar luxation (MPL) presence and grade. A total of 174 joints (68 healthy, 96 grade II MPL, and 10 grade III MPL) from Mini-Pinschers, Yorkshire Terriers, Pomeranians, and Chihuahuas were included in the study. The morphological type of trochlear dysplasia (TD) was evaluated on axial scans and 3D reconstruction images according to the four-type classification of Déjour, sulcus angle, trochlear depth, and lateral/medial inclination angles. Of all 174 joints, 140 had trochlear dysplasia—all joints with MPL (n = 106) and 50% of healthy joints (n = 34). The classification of Déjour for trochlear dysplasia types (A, B, C, and D) corresponds to the morphology of the femoral trochlea in the studied small breeds of dogs. The results demonstrated three types of trochlear dysplasia according to Déjour: most commonly, type A, followed by type C, and most infrequently, type D. The Déjour type B was an incidental finding. The large proportion of clinically healthy joints with TD (50%) emphasises the significance of early trochlear morphology evaluation for the orthopaedical health of dogs from susceptible breeds, especially in female breeders.

## Simple Summary

Trochlear dysplasia is a complex three-dimensional deformity, recognised as the primary contributor to medial patellar luxation in dogs from small breeds, but no studies have investigated its prevalence in the general canine population. Computed tomography allowed for the reliable identification of the different trochlear dysplasia types on axial scans and three-dimensional reconstructed images. The classification of Déjour for trochlear dysplasia types (A, B, C, and D) is appropriate for the description of the abnormal morphology of the femoral trochlea in small breeds of dogs. Mini-Pinschers, Yorkshire Terriers, Pomeranians, and Chihuahuas demonstrated three types of trochlear dysplasia according to Déjour: most commonly, type A, followed by type C, and most infrequently, type D. The Déjour type B was an incidental finding. The study emphasises the role of diagnostic imaging in the investigation of bony stabilisers for orthopaedical clinical decision-making in dogs.

## 1. Introduction

Medial patellar luxation is a common orthopaedic problem in dogs from small breeds. Most cases are considered developmental with anatomical deformities compromising the stifle extensor mechanism—reduced anteversion angle, distal external femoral torsion, excessive distal femoral varus, tibial tuberosity medialisation, patella alta, and shallow trochlear groove [1]. Studies have demonstrated that abnormal trochlear morphology is one of the most important factors predisposing dogs to patellar luxation [2,3,4]. Dogs with trochlear dysplasia (TD) are more likely to suffer from patellar luxation, therefore, the early diagnosis of trochlear dysplasia is extremely important [5].

In humans, femoral trochlear dysplasia was described in 1941 [6]. TD is defined as abnormal morphology of the trochlear groove presented with altered normal concave anatomy, reduced depth, and sometimes, abnormal configuration. The grade of TD ranges from shallow to flat and dome-shaped trochlea [7]. The incongruence between trochlear and patellar surfaces has a high influence on patellar tilt and subluxation. The higher the degree of trochlear dysplasia, the higher the risk of instability [8].

On the basis of computed axial tomography (CT) scans, David Déjour created the four-type classification of TD, which is the current gold standard in orthopaedics [9]. This classification is characterised with moderate to near-perfect inter-observer agreement and remains the most widely used to assess trochlear dysplasia [10].

A three-stage classification system for the assessment of abnormal trochlear development in small dog breeds, mild, moderate, and severe trochlear dysplasia, was proposed several years ago on the basis of measuring groove morphometric parameters [2]. In this first study, the shallow trochlear groove and medial femoral condyle’s hypoplasia were accepted as signs of mild and moderate trochlear dysplasia. So far, no attempt to apply the Dejour classification system to stifle joints in dogs has been made. Also, while trochlear dysplasia is recognised as the primary contributor to patellar instability, no studies have investigated its prevalence in the general population. The present retrospective computed tomography study was designed to evaluate the trochlear groove morphology in four small breeds of dogs—Mini-Pinscher, Chihuahua, Pomeranian, and Yorkshire Terrier—and the prevalence of the trochlear dysplasia types according to the classification of Déjour depending on the breed, sex, medial patellar luxation presence, and grade.

## 2. Materials and Methods

### 2.1. Experimental Subjects

The retrospective study was performed from December 2022 to May 2024 in 128 dogs from small breeds aged from 1.5 to 4 years after obtaining informed consent from their owners. Out of them, 94 dogs (33 Mini-Pinschers, 21 Chihuahuas, 33 Pomeranians, and 7 Yorkshire Terriers) were diagnosed with grade II and III MPL according to Singleton. Another 34 dogs (15 Mini-Pinschers, 6 Chihuahuas, 6 Pomeranians, and 7 Yorkshire Terriers) evaluated to be orthopaedically healthy following clinical and CT examination were used as controls. The total number of investigated joints was 174—68 healthy joints without MPL and 106 joints with patellar luxation (96 joints with grade II and 10 joints with grade III MPL).

The orthopaedic exam consisted of a patellar displacement test, a dancing patella test, and the palpation of hindlimbs. Criteria for the inclusion of dogs/stifles with patellar luxation in the study comprised dogs from small breeds only (Mini-Pinscher, Chihuahua, Pomeranian, and Yorkshire Terrier), the type of patellar luxation (only medial) and grade of MPL (only grade II and III), and positive patellar displacement and dancing patella tests. Exclusion criteria were a traumatic origin of the MPL and the presence of accompanying orthopaedic disease. For MPL-free stifles, inclusion criteria were dogs from the same small breeds (Mini-Pinscher, Chihuahua, Pomeranian, and Yorkshire Terrier), a lack of orthopaedic or neurological affections of the musculoskeletal system (negative patellar displacement and dancing patella tests, symmetrical femoral and gluteal muscles, a lack of worn nails or trophic ulcers suggesting proprioceptive dysfunction, and normal patellar and withdrawal reflexes).

### 2.2. Computed Tomography Studies

After deep i.m. sedation with 0.075 mg/kg medetomidine hydrochloride (Dorbene vet^®^, 1 mg/mL, Syva, León, Spain) and 7.5 mg/kg ketamine hydrochloride (Anaket^®^, 100 mg/mL, Richter Pharma, Wels, Austria), CT images were obtained in a distoproximal direction, with the dog in dorsal recumbency with extended pelvic limbs (OFA-view).

CT scans of the femoral trochlea were performed using a 32-slice CT scanner (Somatom Go Now, Siemens Healthcare GmbH, Erlangen, Germany) in a helical mode. Detector slice thickness was 1 mm; tube voltage: 120 kV; tube rotation time: 0.8–1 s; and pitch: 0.6–1 with tube currents adjusted according to the size of the patient. The reconstructed slice thicknesses and increments were between 1 mm and 3 mm, allowing the creation of continuous 3D CT data using a bone algorithm. DICOM images were imported into Syngo Via View&Go software (Syngo CT VA30, Siemens Healthcare GmbH, Erlangen, Germany).

**Figure 1:**
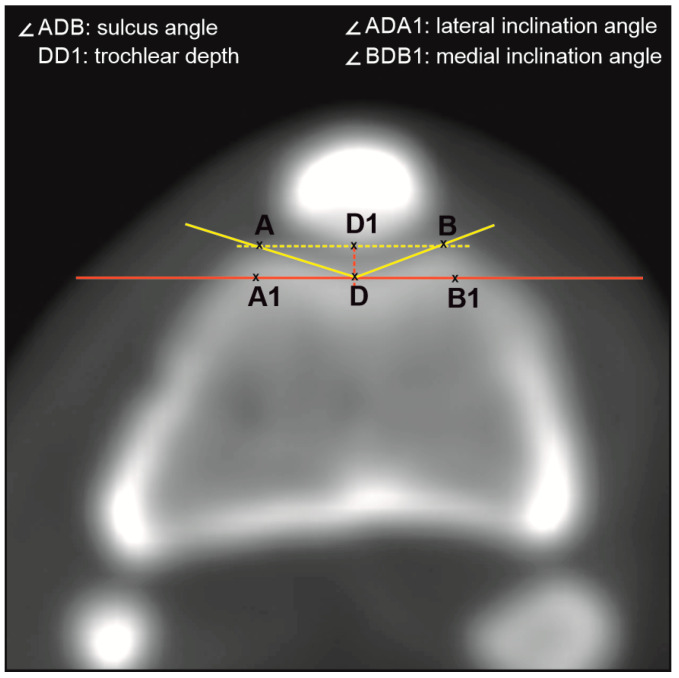
Measurements of the sulcus angle [11], lateral and medial inclination angles [12], and trochlear groove depth [13]. AB: line connecting the lateral (A) and medial (B) femoral condyles; D: sulcus bottom; DD1: perpendicular to AB from the sulcus bottom.

For the measurements, the femoral trochlea axial scan at the level of the “Roman Arch” at the top of the femoral condyle at the deepest point of the femoral trochlea was used (Figure 1). According to Brattstorm [11], the sulcus angle (∠ADB) was formed by lines connecting the highest points of lateral (A) and medial (B) femoral condyles with the deepest point of the trochlear groove (D). The lateral (∠ADA1) and medial (∠BDB1) trochlear inclination angles were measured by the method of Laurin et al. [12]. They are formed by the lines tangential to the posterior condyle (A1B1) passing from the deepest point of the trochlear groove (D) to the lateral (A) and medial (B) condyles. The depth of the trochlear groove (DD1) was measured according to Pfirrmann et al. [13] as the distance between the deepest point of the trochlear groove (D) and the point (D1) of the interception of the line passing through the trochlear facets (AB) and the perpendicular raised from the trochlear bottom to line AB.

### 2.3. Evaluation of Trochlear Dysplasia Type

**Figure 2:**
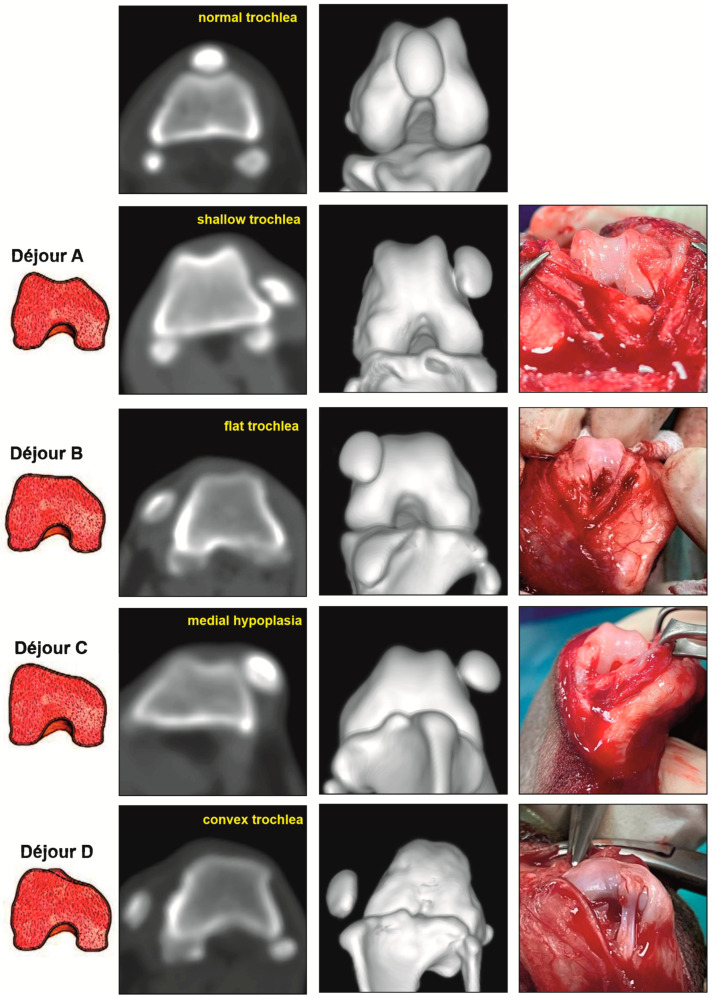
Illustration of morphological types of trochlear dysplasia in dogs on axial CT scans, three-dimensional reconstruction images, and intraoperative views.

The morphological type of femoral trochlear dysplasia on axial CT scans and 3D reconstructed images was determined according to the four-type classification of Déjour [9] and trochlear groove measurements (Figure 2).

On axial scans, the trochlear groove in type A trochlear dysplasia was shallow but with a regular shape. The sulcus angle exceeded 125°. Type B trochlear dysplasia corresponded to a flat trochlear groove. In type C TD, the groove was shallow, with marked medial condylar hypoplasia and normal or hyperplastic lateral condyle, whereas joints with type D trochlear dysplasia were outlined with a convex shape of the groove.

### 2.4. Statistical Analysis

Data are expressed as numbers (percentages) for categorical variables and medians (interquartile ranges) for continuous variables. The comparisons between the groups were performed with the chi-square and Friedman non-parametric tests (MedCalc Software 15.8, Ostend, Belgium).

## 3. Results

**Figure 3:**
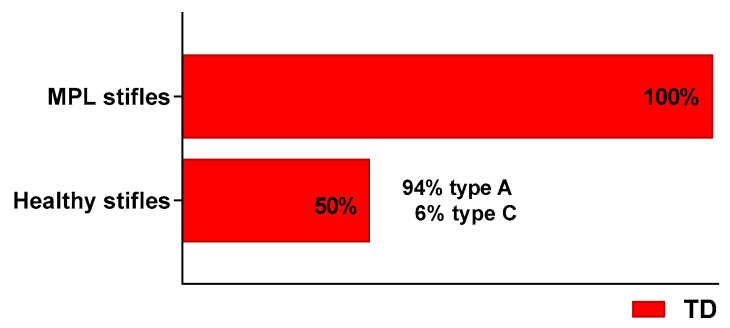
Prevalence of trochlear dysplasia in healthy stifles and stifles with patellar luxation (MPL).

Out of all 174 joints, 140 were diagnosed with various trochlear dysplasia types. This number included all joints with patellar luxation (n = 106) and 50% of the 68 healthy control joints—32 joints (94%) with TD type A and 2 joints (6%) with TD type C (Figure 3).

**Figure 4:**
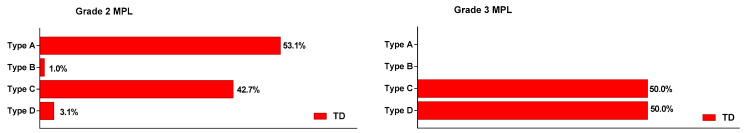
Prevalence of trochlear dysplasia types in stifles with grade II and grade III medial patellar luxation (MPL).

Among the stifle joints with MPL, type A trochlear dysplasia was the most common—found in 51 joints, followed by type C (46 joints) and type D (8 joints). Type B trochlear dysplasia was observed in only one stifle joint with grade II MPL. Trochlear dysplasia of type A was diagnosed in the majority of joints with grade II MPL (51 joints; 53.1%) followed by type C (41 joints; 42.7%) and type D (3 joints; 3.1%). The stifle joints with grade III MPL demonstrated trochlear dysplasia only of types C and D—five joints (50%) of each of the two types (Figure 4).

**Figure 5:**
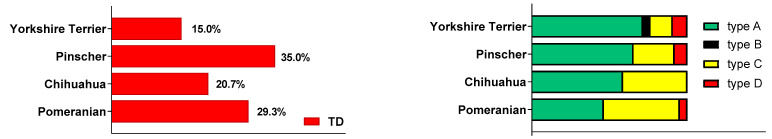
Prevalence of trochlear dysplasia types in relation to the breed.

From all studied joints, the highest percentage of TD was observed in Mini-Pinschers followed by Pomeranians, Chihuahuas, and Yorkshire Terriers, but the inter-rate differences were not statistically significant (*p* = 0.0896). Type A dysplasia predominated in all breeds except for Pomeranians, where type C was the most frequently seen (Figure 5). Among healthy control joints, the breed-wise distribution showed the highest rate of TD in Yorkshire Terriers—11/17 joints (64.7%)—followed by Chihuahuas (5/8 joints; 62.5%). In joints from Mini-Pinschers and Pomeranians without MPL, the proportion of joints affected with TD was 40.6% (13/32 joints) and 45.5% (5/11 joints), respectively.

**Figure 6:**
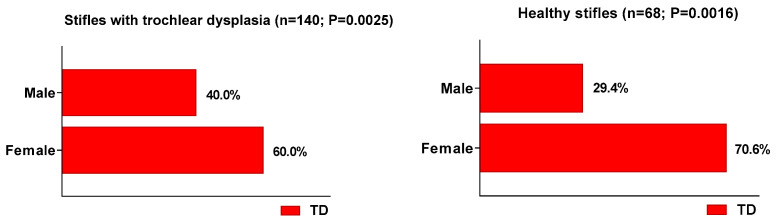
Sex-associated prevalence of trochlear dysplasia in all 140 TD stifle joints (**left**) and among 68 healthy control stifle joints (**right**).

On the basis of all joints with trochlear dysplasia, the abnormal morphology was statistically significantly (*p* = 0.0025) more common in stifles from female dogs than in those from males. An even more pronounced sex-related difference (*p* = 0.0016) was observed between joints from females and males classified as orthopaedically healthy (Figure 6). As far as the specific type of TD was concerned, out of the 48 joints diagnosed with type C trochlear dysplasia, those from females were twice as numerous (n = 32) compared to those of males (n = 16; *p* = 0.0022).

The results from trochlear groove measurements in joints without trochlear dysplasia and joints with various TD types are presented in Table 1. The sulcus angle (SA) in joints without TD was smaller than those with TD types A, B, C, and D (*p* < 0.001). Statistically significant differences in median SA values were proven among the various TD types as well. In type A TD, the SA was considerably smaller (*p* < 0.001) vs. those of types C and D.

The trochlear groove depth of morphologically normal joints was greater (*p* < 0.001) than that of dysplastic grooves from the other types. The depth of joints with type D dysplasia was zero.

Statistically significant differences were found for the lateral inclination (LTI) and medial inclination (MTI) angles of non-dysplastic joints and those with trochlear dysplasia type A and type C (*p* < 0.001). In type D dysplasia, the median LTI and MTI values were zero (Table 1).

## 4. Discussion

Similar to results from diagnostic imaging studies in humans [14], the axial CT scans and 3D images of the trochlear groove of small-breed dogs clearly visualise the changes in its normal morphology—shallow, flattened, asymmetrical (due to hypoplasia of the medial and hyperplasia of the lateral condyles), and convex (dome-shaped)—and allow for the identification of the Déjour morphotypes of trochlear dysplasia (A, B, C, and D).

Unlike veterinary medicine literature, several human medical studies have documented the prevalence of the different morphological types of trochlear dysplasia. Déjour et al. [15] compared radiographs and tomograms of patients with patellar instability (PI) and healthy stifles and reported that 85% of PI and 3% of control subjects had trochlear dysplasia. Similar rates were reported by Bollier and Fulkerson [16]—between 0.7 and 2% in healthy joints and up to 85% in patients with PI. Another study reported TD prevalence in humans of about 6% and a close association between trochlear dysplasia not only with PI but also with patellofemoral joint pain [17]. The most recent published data provided evidence for a general prevalence of TD of 4.5%, with more cases in Asian and Caucasian races [18].

The present study provides, for the first time, computed tomography data for a significant prevalence rate of trochlear dysplasia in clinically healthy small-breed dogs—50%—with a predominance of type A according to Déjour’s classification. This finding strongly suggests that trochlear dysplasia is a major but not the only factor contributing to clinical patellar luxation. Thus, despite being born with trochlear dysplasia, a dog will most probably remain asymptomatic if no changes in soft tissue stabilisers or bone deformities altering stifle biomechanics are present. Another feasible hypothesis is the different degrees of changes within the same TD morphotype. For example, the analysis of measured parameters for joints with Déjour type A TD shows that joints with clinically manifested MPL had significantly lower trochlear depth (*p* < 0.001)—a median (IQR) of 1.2 (1.1–1.4) mm compared to asymptomatic Déjour type A joints, with a median (IQR) of 2.0 (1.35–2.5) mm. The median (IQR) of trochlear depth in joints without trochlear dysplasia was determined to be 2.2 (2.0–3.0), e.g., closer to values of asymptomatic joints (Table 1). It should be noted that there were no statistically significant differences in LTI and MTI, meaning that femoral condyles for MPL-free and MPL grade 2 joints diagnosed with Déjour type A were symmetrical.

The dog breed and the patellar luxation occurrence are closely related [19]. One study reported that Pomeranians and Chihuahuas were most frequently affected with MPL [19], whereas another affirmed that patellar luxation was the most prevalent among Yorkshire Terriers, Poodles, Chihuahuas, and Boston Terriers [20]. In the present retrospective study, trochlear dysplasia was encountered in all studied breeds with the highest rates in Mini-Pinschers (35%) and Pomeranians (29%). Unlike three of the studied breeds, the predominant trochlear dysplasia type in Pomeranians was type C. It may be presumed that the selective breeding of Pomeranians, leading to genetic abnormalities and patellar luxation in particular, may be involved. Genomic analyses of the dogs from this breed have shown that a region on chromosome 7 was possibly associated with MPL development [21].

The present study provided evidence that trochlear dysplasia was statistically significantly more common in female dogs, especially among stifle joints classified as morphologically normal (70.6% from female and 29.4% from male dogs). In small-breed dogs, the medial trochlear condyle is significantly shorter in females than in males [22]. The characteristic medial condyle hypoplasia in females may provide an explanation as to the fact that type C trochlear dysplasia was twice as common in joints from female dogs. In humans, the increased prevalence of PI in women is usually attributed to anatomical differences between sexes, especially with patellofemoral alignment. These differences include higher rates of trochlear dysplasia, patella alta, increased Q angle, and soft tissue imbalances [23,24]. In agreement with our results, the reported Pearson correlation between dysplasia and sex was moderate and positive in Caucasian and Asian female patients (*p* = 0.01) but not in the African cohort (*p* = 0.40). Middle Eastern male patients demonstrated more dysplastic values than their female counterparts; however, this was without a significant association with sex (*p* = 0.15) [18].

The trochlear dysplasia is a complex three-dimensional deformity, so multiple diagnostic imaging quantitative and qualitative criteria for its grading are published [25]. After 1990, a total of 46 individual parameters for the description of TD are introduced [26]. The main quantitative morphological criteria are the sulcus angle, the tibial tuberosity–trochlear groove distance, and trochlear depth and inclination angles [27]. A systematic analysis of imaging data from 30 different parameters in 3036 patients performed by Saccomanno et al. [28] has shown that the sulcus angle, trochlear depth, and Dejour type were the most popular parameters for reliable trochlear dysplasia evaluation.

The four-type classification of Déjour was created to assist the categorisation of trochlear dysplasia (A, B, C and D) in order to assist surgical decision-making regarding different morphological groove abnormalities [29]. This classification turned out to be applicable to the description of the morphological types of TD in dogs. In the literature, it is still disputable which of all types of TD according to Déjour may be classified as severe trochlear dysplasia. In this connection, some researchers have simplified the original four-type classification to two types, namely, mild trochlear dysplasia (types A and C) and severe TD (types B and D) [30]. Our study, involving 174 stifle joints from 128 small-breed dogs, also observed the most severe changes in type D dysplasia, confirmed by great and statistically significant differences in sulcus angle, trochlear depth, and inclination angle values. The latter three indices could not be quantified because of the dome-shaped trochlea.

The trochlear dysplasia in orthopaedically healthy stifle joints was from the mild type (94% type A and 6% type C). In humans, Biedert and Bachmann [31] reported that the majority (83.4%) of dysplastic trochleae evaluated by axial MRI had elevated trochlear floor and normal lateral condylar height, and 16.6% of patients with trochlear dysplasia had reduced lateral but normal central trochlear height.

Medial patellar luxation in dogs is accompanied by anatomical deviations affecting the entire pelvic limb. Its severity and the extent of deformities are graded by the 4-point system of Singleton [32]. In this study, the most commonly encountered TD types in the joints with grade II MPL were types A and C, whereas the TD morphotypes in joints affected with grade III MPL were only from types C and D. This finding supported the close relationship between the extent of morphological trochlear abnormalities and patellar luxation grade.

Although providing an insight into the origins of medial patellar luxation in dogs, several limitations of this study should be mentioned: the low number of evaluated healthy and MPL stifles, the inclusion of four small dog breeds only, the lack of data on stifles with grade IV MPL, and the lack of data about the inter-rater agreement of the applied classification.

## 5. Conclusions

The morphology of the femoral trochlea and the different types of trochlear dysplasia can be clearly and reliably identified on computed tomography scans and three-dimensional reconstructed images. The trochlear dysplasia types according to the classification of Déjour are appropriate for the description of the abnormal morphology of the femoral trochlea in small breeds of dogs. On the basis of results, Mini-Pinschers, Yorkshire Terriers, Pomeranians, and Chihuahuas demonstrated three morphological types of trochlear dysplasia according to Déjour: most commonly, type A, followed by type C, and most infrequently, type D. The Déjour type B was an incidental finding.

In this study, a large proportion of clinically sound stifles had trochlear dysplasia, which confirms the significance of the early evaluation of trochlear morphology for the orthopaedical health of dogs from predisposed small breeds, especially in female breeders. Furthermore, the presence of trochlear dysplasia in all joints with medial patellar luxation emphasises the thorough investigation of bony stabilisers in canine orthopaedical clinical decision-making.

## Figures and Tables

**Table 1:** Trochlear angles and trochlear depth in healthy canine stifle joints and joints with various types of trochlear dysplasia (TD). Data are presented as medians (interquartile ranges).

	Joints Without TD (n = 34)	TD Type A (n = 83)	TD Type B (n = 1)	TD Type C (n = 48)	TD Type D (n = 8)
Sulcus angle, °	120 (118–121)	130 (127–134) *	149	139.5 (135–144.5) *	180 (156–180) *
	P_A—C_ < 0.001; P_A—D_ < 0.001; P_C—D_ < 0.001				
LTI, °	29 (27–31) *	26 (24–28) *	1	24.5 (23–27) *	0 (0–1)
	P_A—C_ < 0.05				
MTI, °	28 (27–29) *	25 (23–28.8) *	1	20 (19–22) *	0 (0–0.5)
	P_A—C_ < 0.001				
Trochlear depth, mm	2.2 (2.0–3.0) *	1.4 (1.1–1.8) *	0.1	1.0 (0.8–1.2) *	0 (0–0)
	P_A—C_ < 0.001				

n—number of joints; LTI—lateral trochlear inclination; MTI—medial trochlear inclination; * statistically significant differences in TD types vs joints without TD (*p* < 0.001). Subscripts A, C, and D indicate the level of statistical significance between measures of respective TD morphotypes.

## Data Availability

The data presented in this study are available upon request from the corresponding authors.
